# Erosive cheilitis as an early manifestation in DRESS syndrome

**DOI:** 10.1002/ccr3.5123

**Published:** 2021-11-22

**Authors:** Wahbi Ben Salha, Eya Moussaoui, Lamia Oualha, Jihed Anoun, Nabiha Douki

**Affiliations:** ^1^ Department of Dental Medicine Dental Faculty of Monastir SAHLOUL Hospital (Sousse) University of Monastir Monastir Tunisia; ^2^ Laboratory of Oral Health and Maxillofacial Rehabilitation (LR12ES11) University of Monastir Monastir Tunisia; ^3^ Department of Internal Medicine Faculty of Medicine of Sousse SAHLOUL Hospital (Sousse) Sousse Tunisia

**Keywords:** cutaneous adverse reaction, DRESS syndrome, drug reaction, erosive cheilitis, oral lesions

## Abstract

Drug reaction with eosinophilia and systemic symptoms (DRESS) is a distinct part of severe cutaneous adverse reactions (SCARs). It is characterized by fever, rash, hematologic abnormalities, lymphadenopathy, or/and different degrees of visceral organ involvement. Its diagnosis is particularly challenging due to the variability of its clinical presentations and its long latency period (2–6 weeks). Allopurinol, an uric acid‐lowering drug, has been incriminated in several cases of allopurinol‐induced DRESS syndrome. Through this paper, we present a case of allopurinol‐induced DRESS syndrome with initial oral mucosal involvement. A 69‐year‐old female patient presented with an erosive cheilitis that started 1 week prior to his presentation. The cheilitis was associated with maculopapular rash and fever. She started taking allopurinol, as treatment of Gout, 6 weeks before hospitalization. The histologic findings obtained from skin biopsy were consistent with a toxic drug reaction. A complete blood count (CBC) showed a moderate eosinophilia. Alteration of renal function was also noted, and the diagnosis of allopurinol‐induced DRESS syndrome was made. Systemic corticosteroid therapy was therefore started. The patient completely recovered and had been healthy for 3 years before developing a recurrence after re‐challenge with allopurinol.

## Graphical abstract

DRESS syndrome is a rare and potentially life‐threatening severe cutaneous adverse reaction that should be quickly diagnosed to guide its management, that implies a perfect knowledge of its clinical features and symptoms. In this context, cheilitis can be initial manifestation of this syndrome as reported in this paper.

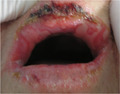

## 1 INTRODUCTION

Adverse cutaneous drug reactions involve a variety of clinical manifestations, ranging from minor skin rashes to fatal hypersensitivity reactions called severe cutaneous adverse reactions (SCARs). SCARs include Steven‐Johnson syndrome, toxic epidermal necrolysis, acute generalized exanthematous pustulosis, and DRESS syndrome.

Stevens‐Johnson syndrome (SJS) and toxic epidermal necrolysis (TEN) are critical and life‐threatening cutaneous reactions characterized by blisters, extensive detachment of epidermis, and mucosal erosions.^1^ Stevens‐Johnson syndrome and TEN are considered to be a single disease entity with common mechanisms.^2^ Immune disorders triggered by medications and infections, such as by the herpes simplex virus or Mycoplasma, are the main causes.^3^ Stevens‐Johnson syndrome and TEN are differentiated by the percentage of skin detachment area^4^ (Table 1).

**TABLE 1:** Classification of SJS/TEN

Diagnosis	Skin detachment area (%)
SJS	10
SJS/TEN overlap	10–30
TEN	>30

Abbreviations: SJS, Stevens‐Johnson syndrome; TEN, toxic epidermal necrolysis.

Acute generalized exanthematous pustulosis (AGEP) represents a rare severe cutaneous adverse reaction (SCAR) characterized by the rapid development of nonfollicular sterile pustules on an erythematous background affecting large areas of the body. It is usually related to medication administration, and the duration between drug exposure to reaction onset is typically within 48 h.^5^

Drug reaction with eosinophilia and systemic symptoms (DRESS), also known as drug‐induced hypersensitivity syndrome (DIHS), is a severe cutaneous adverse reactions (SCAR) characterized by fever, rash, hematologic abnormalities, lymphadenopathy, and different degrees of visceral organ involvement.^6^ The liver is the visceral organ most commonly involved.^7^ Clinical features of this involvement range from mild elevation of liver enzymes to acute hepatic failure.^8^ Kidney is the second most involved organ in this disease where we noted hematuria, nephritis, and acute renal failure in the severe form.^9^

Lungs are the third most involved organ, present in 6, 7, to 10% of patients with DRESS syndrome. Clinical presentations include mild cough, pneumonitis, and acute respiratory distress syndrome in the advanced stage.^9^

Cardiac involvement is also reported in this syndrome. Clinical features include pericarditis in the mild form, carditis in moderate form, and congestive heart failure in critical forms.^9^

GIT involvement is among the lesser‐known manifestations of the DRESS syndrome. It includes pancreatitis, gastritis, esophagitis, enteritis, and colitis. Fulminant type 1 diabetes mellitus (T1DM), autoimmune type1 diabetes mellitus (T1DM), and type 2 diabetes mellitus (T2DM) were reported as a late autoimmune sequela due to pancreatic injury. Despite the rarity of these gastrointestinal entities in Dress, their development is associated with increased mortality.^10^

In contrast with other SCARs, the unique features of DRESS Syndrome are the involvement of various internal organs and the delayed onset of clinical manifestations after administration of the culprit drugs (generally 2–8 weeks).^10^, ^11^ The medications reported to be most frequently associated with DRESS syndrome are aromatic anticonvulsants, dapsone, sulfasalazine, and allopurinol.^12^

This condition, which can lead to death in 10% of cases,^13^, ^14^ is commonly overlooked and misdiagnosed.^15^

Through this paper, a case of allopurinol‐induced DRESS syndrome with initial oral mucosal involvement is presented while emphasizing the importance of early diagnosis to achieve the appropriate management.

## 2 CASE REPORT

A 69‐year‐old female patient was referred to our department by the internal medicine department for lesions of the oral mucosa.

Her medical history revealed diabetes mellitus (type 2) for 17 years, hypertension for 10 years, dyslipidemia, and Gout disease. She had a surgical history of coronary artery bypass surgery 11 years earlier and cholecystectomy 17 years earlier. Medications involved metformin, Glibenclamide, Captopril, Isosorbide dinitrate, Propranolol, Fluvastatin, Aspirin, and Colchicine. She started taking Allopurinol^®^ 6 weeks before hospitalization for a gout flare‐up. No history of drug hypersensitivity reactions was identified.

The patient was a housewife living with her husband in an urban setting, mother of 6 daughters, and had no prior travel history. She had no history of smoking, alcohol consumption, or illicit drug use. No allergies were identified.

A week before her hospitalization, the patient developed fever associated with chills, night sweats, and malaise. The following day, the patient noticed bluish spots on the lower limbs along with a significant labial edema. She consulted the emergency department where she had an unspecified symptomatic treatment, without improvement. Then, the patient consulted a dermatologist who prescribed corticosteroid (prednisolone) as a mouthwash and referred the patient to the internal medicine department where she was hospitalized.

**FIGURE 1:**
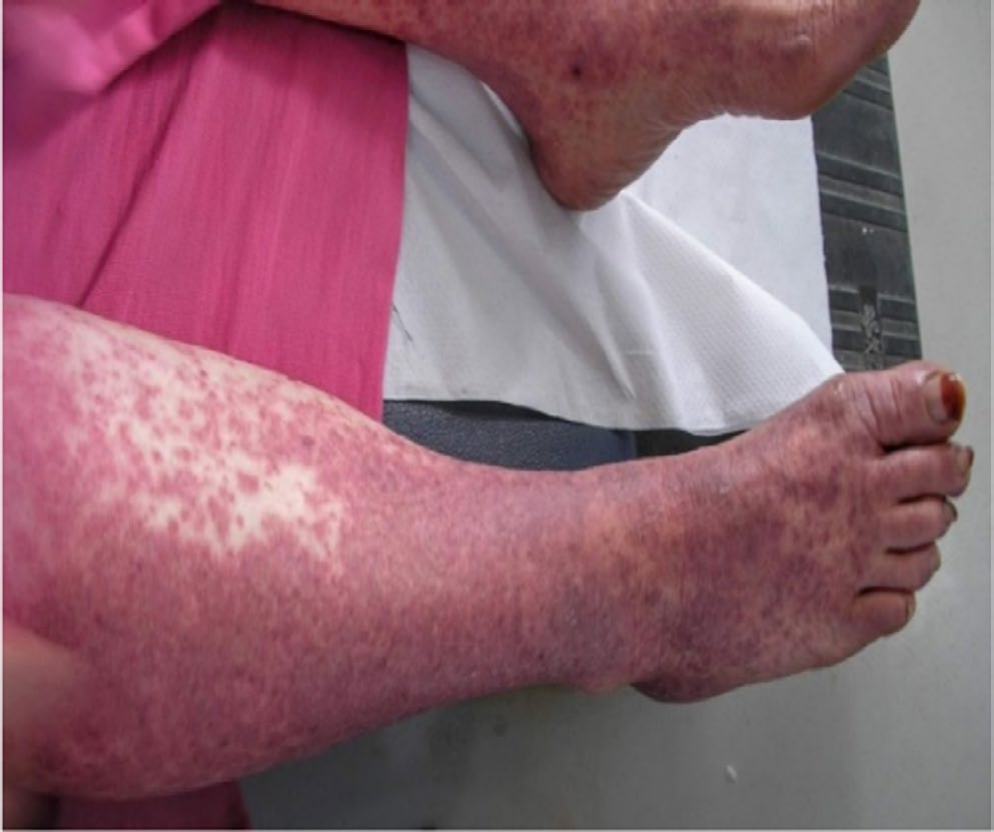
Rash on the lower extremities

**FIGURE 2:**
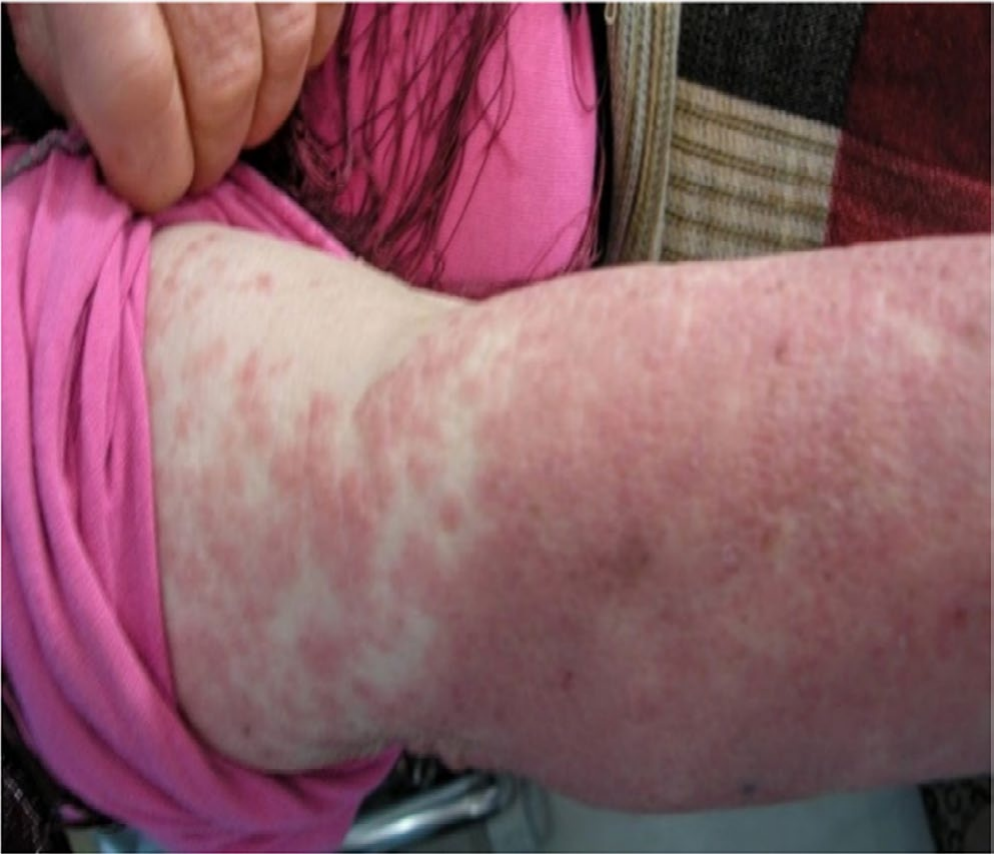
Rash on the upper extremities

On the first day of admission to the internal medicine department, the patient was conscious and well‐oriented. The initial recorded temperature was 38.7°C, blood pressure was 120/70 mmHg, pulse was 67 beats/minute, and weight was 72 kg. Physical examination showed the presence of a confluent erythematous maculopapular rash, diffused all over the body (feet, legs, stomach, chest, and back), and sparing the face, scalp, palms, and soles. (Figures 1 and 2). Nikolsky's sign was negative. No lymphadenopathy was present. On auscultation, the chest was clear on both sides. The patient's heart had a regular rate and rhythm. The remainder of the examination was without abnormalities.

**FIGURE 3:**
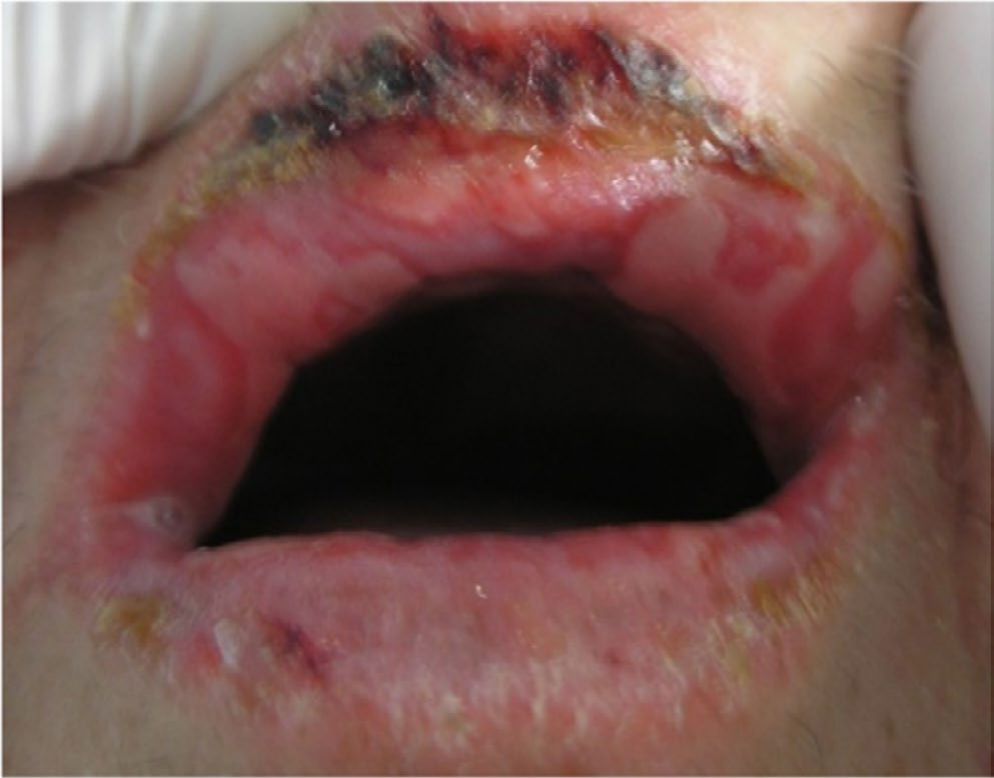
Crusting on the lips with confluent ulcerations

In the department of dental medicine, oral examination showed the presence of a painful erosive cheilitis, crusty lesions on both lips, and confluent ulcerations across the labial mucosa (Figure 3). These aspects were reminiscent of those seen in some bullous drug eruption (erythema multiforme, Stevens‐Johnson syndrome, etc.) but the Nikolsky sign was negative. Antibiotics (amoxicillin 2 g per day) were prescribed to avoid infection of the lesions. Local corticosteroid therapy (Prednisolone as a mouthwash), analgesic, and chlorhexidine‐based mouthwash were also prescribed. Oral biopsy was scheduled.

Complete blood count (CBC) showed a normal number of white blood cells (WBCs) of 10.28*10^3^/mm^3^ with 9.4% lymphocytes, 11.3% monocytes, and 16.1% (1.65*10^3^/mm^3^) eosinophils, corresponding to a moderate eosinophilia. C‐reactive protein was elevated at 13 mg/L. Results of tests for serum electrolytes, hemoglobin, hematocrit, and sedimentation rate were normal.

Serology for hepatitis B and hepatitis C was negative. Urine and blood cultures were also negative. Uric acid level was high. Similarly, high levels of Serum glucose and triglyceride were noted.

**FIGURE 4:**
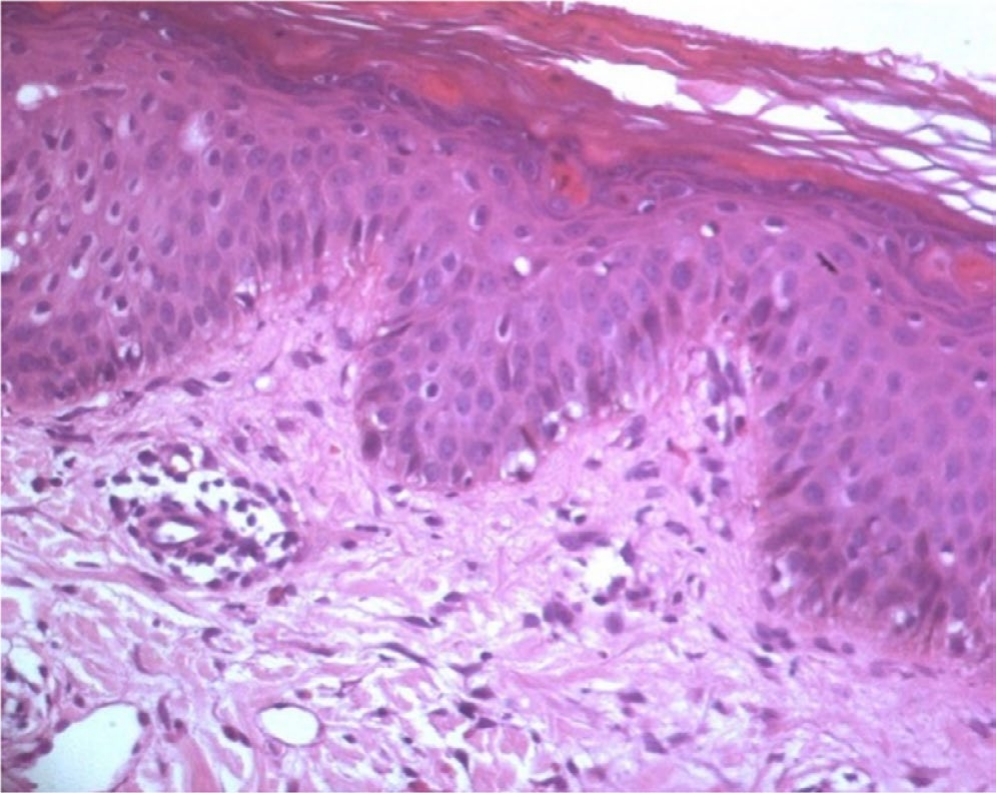
Histological section of the skin showing necrotic keratinocytes and a subepidermal perivascular inflammatory infiltrate, consisting of lymphocytes and eosinophils

Skin biopsy was performed and it showed a subepidermal inflammatory infiltrate, consisting of lymphocytes and eosinophils (Figure 4). This histological aspect was in accordance with a toxic drug reaction.

Based on the patient's history, clinical presentation, and biological tests, diagnosis of cutaneous adverse drug reaction was made. Systemic corticosteroid therapy (oral prednisolone) was therefore started. The most likely etiology was allergic response to allopurinol.

On the seventh day of admission, the patient had an alteration in her renal function with creatinine level increased to 116 μmol/L blood. Glucose level was raised from 7.0 to 9.8 (mmol/L). Liver function tests showed an albumin level of 31 g/L, an abnormal coagulation panel with an international normalized ratio (INR) of 1.3, aspartate aminotransferase (AST) levels of 34 IU/L, and a slightly elevated alanine aminotransferase (ALT) levels of 47 IU/L.

Diagnosis of drug reaction with eosinophilia and systemic symptoms (DRESS) was therefore made.

**FIGURE 5:**
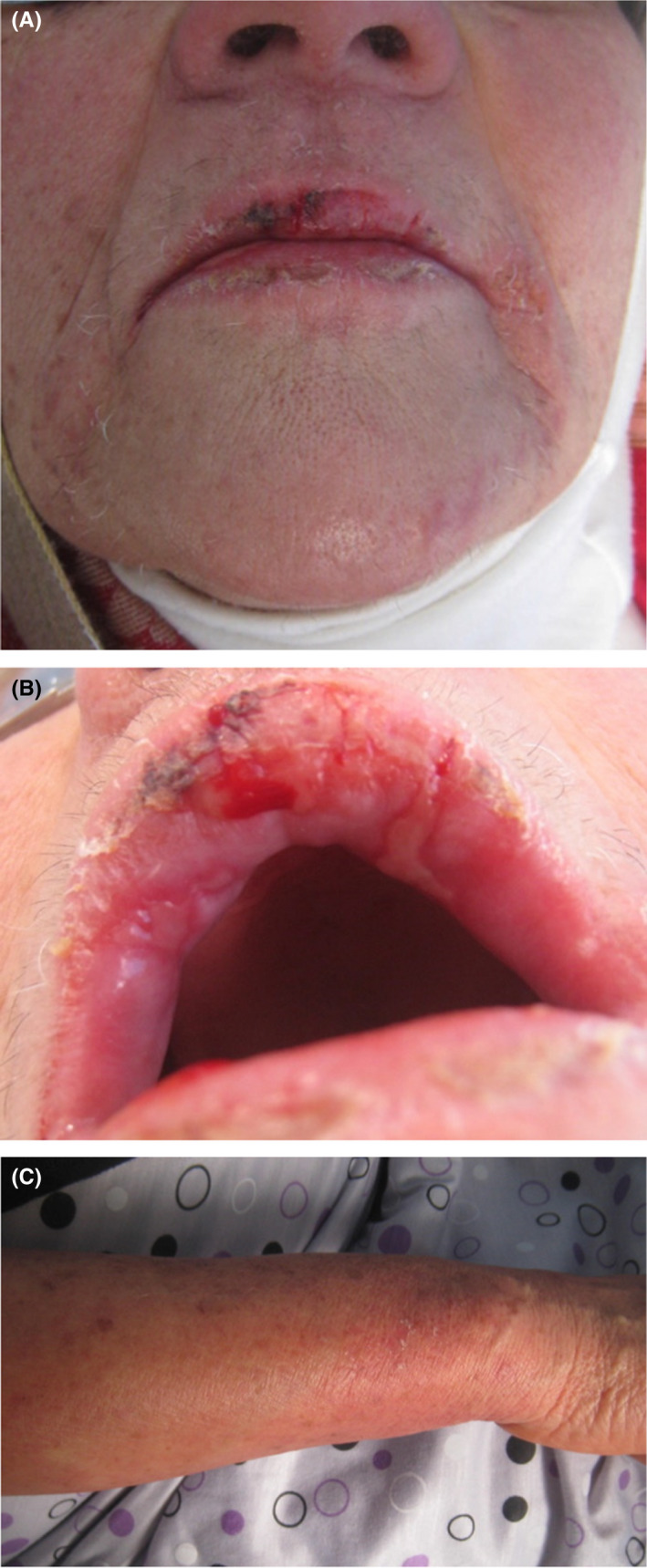
An improvement in both oral (a,b) and cutaneous (c) lesions was noted 5 days after corticosteroid therapy

Nine days after corticosteroid therapy, a spectacular improvement in both cutaneous and oral lesions was noted. (Figures 5a–c).

Three years later, the patient was rehospitalized with similar cutaneous lesions after self‐medication using allopurinol. This episode started 10 days after drug administration with fever and maculopapular rash. Oral mucosa was not involved. This second form was quickly diagnosed and managed with immediate withdrawal of the culprit medication and administration of corticosteroid therapy as in the first episode.

## 3 DISCUSSION

Severe cutaneous adverse reactions (SCARs) are a class of life‐threatening adverse drug responses affecting the skin and the mucosal surfaces (oral, genital, ocular, etc.). They can cause severe damages to internal organs in more critical cases.^16^

Drug reaction with eosinophilia and systemic symptoms (DRESS) is a distinct part of SCARs, and it constitutes a challenge with regard to diagnosis, management, and treatment.^16^

The pathophysiology of DRESS is not yet fully understood. It has been suggested that certain drugs may cause hypersensitivity reactions in patients with genetic or acquired mutations in the drug metabolism pathways, due to abnormal production and detoxification of their active metabolites. Allopurinol was introduced in 1963 as a uric acid‐lowering drug. Its mechanism of action involves its conversion to oxypurinol after being absorbed. It is speculated that excessive oxypurinol can cause tissue damage, trigger immune response, and produce antibodies against tissue components. Others have invoked cell‐mediated immunity.^17^, ^18^ Virus reactivation, especially human herpesvirus 6 (HHV‐6), has been considered an important factor in the pathogenesis of DRESS syndrome.^19^

Several diagnostic criteria have been utilized to standardize DRESS diagnosis. Bocquet et al. were the first to propose criteria for the diagnosis of DRESS in 1996^20^ [Table 2]. In 2007 [Table 1], the Registry of Severe Cutaneous Adverse Reaction (RegiSCAR) group added criteria to diagnose DRESS syndrome and a scoring system to provide a more precise definition. [Tables 3 and 4].^21^ This system tends to be the most widely used and accepted tool. Another set of diagnostic criteria was proposed by a Japanese group^22^ [Tables 5 and 6]. The use of this Japanese model is limited because it requires laboratory measurement of Ig G anti‐HHV6, which is not routinely available. Using the RegiSCAR scoring system, our patient's score was 5, indicating that it was a probable case of DRESS syndrome.

**TABLE 2:** Diagnostic criteria for DRESS as proposed by Bocquet et al

1‐Cutaneous drug eruption
2‐Adenopathy >2 cm in diameter
*Hepatitis (liver transaminases >2 times of normal)
*Interstitial pneumonia or carditis
3‐Hematologic abnormalities eosinophilia >1.5*10^3^/L or atypical lymphocytes.

Note

DRESS is confirmed by the presence of 1, 2, and 3.

**TABLE 3:** Registry of severe cutaneous adverse reaction criteria for diagnosis of DRESS

Hospitalization* Reaction suspected to be drug‐related* Acute rash* Fever >38°C ↑ Enlarged lymph nodes at a minimum of 2 sites Involvement of at least 1 internal ↑ Blood count abnormalities ↑ Lymphocytes above or below normal limits Eosinophils above the laboratory limits Platelets below the laboratory limits.

Note

*Necessary criteria required for making diagnosis.

↑Three out of four criteria are required.

**TABLE 4:** Registry of severe cutaneous adverse reaction diagnosis score for DRESS

Features	No	Yes	Unknown
Fever >38.5°C	−1	0	−1
Enlarged lymph nodes (>2 sites, >1 cm)	0	1	0
Atypical lymphocytes	0	1	0
Eosinophilia			
700–1499 or 10%−19.9%	0	1	
≥1500 or ≥20%		2	
Skin rash			
Extent ≥50%	0	1	
At least2: Edema, infiltration, purpura, scaling	−1	1	
Biopsy suggesting DRESS	−1	0	
Internal organ involvement			
One	0	1	0
Two or more		2	
Resolution in ≥15 days	−1	0	−1
Evaluation of other potential causes (antinuclear antibody, blood culture, serology for HAV/HBV/HVC, chlamydia/mycoplasma)	0	1	0
If none of these positive and >3 are negative			

Note

Final score <2: No case, Final score 2–3: possible case, Final score 4–5: probable case, and final score >5: definite case.

**TABLE 5:** Japanese group's criteria for diagnosis of DRESS/DIHS

Developing maculopapular rash >3 weeks starting with the suspected drug. Prolonged clinical symptoms 2 weeks after discontinuation of the suspected drug Fever >38°C Liver abnormalities (ALT >100 U/L)* Leucocyte abnormalities (at least one present) (a) Leukocytosis (>11*109/L) (b) Atypical lymphocytosis (>5%) (c) Eosinophilia (>1.5*109/L) Lymphadenopathy Human Herpes 6 reactivation

Note

Diagnosis is confirmed by the presence of the 7 criteria (typical DIHS) or the five (1–5) (atypical DIHS). *: This can be replaced by other organ involvement, such as renal involvement.

**TABLE 6:** The patient's score using the RegiSCAR scoring system

Features	No	Yes	Unknown
Fever >38.5°C		0	
Enlarged lymph nodes (>2 sites, >1 cm)	0		
Atypical lymphocytes	0		
Eosinophilia			
700–1499 or 10%−19.9%		1	
≥1500 or ≥20%			
Skin rash			
Extent ≥50%		1	
At least2: Edema, infiltration, purpura, scaling		1	
Biopsy suggesting DRESS		0	
Internal organ involvement			
One		1	
Two or more			
Resolution in ≥15 days		0	
Evaluation of other potential causes (antinuclear antibody, blood culture, serology for HAV/HBV/HVC, chlamydia/mycoplasma)		1	
If none of these is positive and >3 are negative			

Note

Final score = 4>>>>probable case.

Drug history was the key to diagnosis in our case as allopurinol had been incriminated in several cases of allopurinol‐induced DRESS syndrome.^23^

DRESS is still challenging on multiple levels despite the presence of well‐defined criteria. Dermatological involvement presents a notable overlap among the other SCARs, such as Stevens‐Johnson syndrome (SJS), toxic epidermal necrolysis (TEN), and acute generalized exanthematous pustulosis (AGEP). No pathognomonic skin rash pattern for DRESS is available.^24^ Systemic symptoms and negative Nikolsky's sign are two clinical indicators differentiating DRESS from other maculopapular drug eruptions. Skin biopsy is the gold standard for diagnosis. Subepidermal bullae are present in SJS/TEN; however, eosinophilic infiltrate is present in DRESS.

The timing of cutaneous manifestations is also challenging in terms of diagnosis because DRESS and SJS/TEN overlap. Indeed, SJS usually occurs within 1–3 weeks while DRESS occurs within 6 weeks of the drug administration^25^ as observed in our patient.

Other differential diagnoses for DRESS syndrome include acute infections (viral exanthemas, streptococcal, and staphylococcal shock syndrome), autoimmune diseases (hypereosinophilic syndrome, and Kawasaki disease), and neoplastic diseases (lymphomas).^26^

Involvement of the oral mucosa and the vermillion border in DRESS syndrome are frequent. The usually encountered manifestations are nonspecific, including cheilitis,^27^ erosions,^28^ crusting lips,^29^ and edema^22^ as observed in the reported case.

There are no specific treatment guidelines for DRESS syndrome management. The only definitive treatment is to identify and eliminate the culprit drug.^30^ In our case, allopurinol cessation led to healing in 15 days.

In mild forms of DRESS syndrome where cutaneous manifestations are predominant and without significant internal organs involvement, only the cessation of the culprit medication and symptomatic treatment using topical steroids, antihistamines, are sufficient.^10^, ^31^

Immunosuppressive therapy, most often using steroids, is required when visceral organ involvement is present. In 2010, the French Society of Dermatology recommended the administration of systemic corticosteroid in case of organ involvement such as liver (transaminases >5 times upper limit of normal), kidney, lungs, and heart. In this case, prednisone (or equivalents) at a dose equivalent to 1 mg/kg/day can be prescribed.^32^ According to case reports, case series, and retrospective studies, many cases resolved with steroids. Furthermore, following a steroid taper, relapses are common. That's why, corticosteroids are considered the main treatment for severe forms of DRESS syndrome. Nevertheless, randomized controlled trials are lacking, and their administration remains controversial.^33^

Steroid sparing treatments (cyclosporine, cyclophosphamide, intravenous immunoglobulins (IVIG), and plasma exchange) have been employed in cases of steroid failure or contraindications.^29^

Cyclosporine is considered a second line therapy because of its effect on interleukin 5 production (which is required for the development of eosinophilia).^29^

Intravenous immunoglobulins (IVIG) have also been reported as favorable in a few patients with DRESS syndrome and deleterious in others.^32^, ^34^, ^35^

Moreover, benralizumab (IL‐5‐receptor specific humanized monoclonal IgG antibody) has recently been successfully used in treatment of some cases of DRESS syndrome.^36^

## 4 CONCLUSION

Drug reaction with eosinophilia and systemic symptoms (DRESS) is a life‐threatening condition that should always be suspected in patients with fever, rash, and in those with a history of high‐risk drug use taken within the past 8 weeks. Oral mucosa lesions are frequently present. Early diagnosis and withdrawal of the culprit medication are the cornerstone of the appropriate management.

## CONFLICT OF INTEREST

The authors declare that they have no conflict of interest.

## AUTHOR CONTRIBUTIONS

The author contributed equally to this work.

## ETHICAL APPROVAL

This article does not contain any studies involving human participants performed by any of the authors.

## CONSENT

Witten informed consent was obtained from the patient to publish this report in accordance with journal's patient consent policy.

## Data Availability

The data used to support the findings of this study are included within the article.
